# Effectiveness of blended pedagogy for radiographic interpretation skills in operative dentistry - a comparison of test scores and student experiences at an undergraduate dental school in Pakistan

**DOI:** 10.1186/s12909-024-05062-5

**Published:** 2024-01-22

**Authors:** Marium Iqbal, Jacqueline Maria Dias, Amber Sultan, Hussain Ahmed Raza, Laeeq-uz Zaman

**Affiliations:** 1 Department of Operative Dentistry, Jinnah Medical and Dental College, Karachi, Pakistan; 2 Department of Nursing, College of Health Sciences, University of Sharjah, Sharjah, United Arab Emirates; 3 Department for Educational Development, The Aga Khan University, Karachi, Pakistan; 4 Question Bank, Jinnah Sindh Medical University, Karachi, Pakistan

**Keywords:** Blended pedagogy, CoI instrument, Student satisfaction, Dentistry, dental radiology

## Abstract

**Background:**

Utilizing Blended pedagogy (BP) in radiographic skills may prove to be an effective teaching strategy. However, studies on the use of BP in dentistry are quite limited in Pakistan, where teaching has mostly been via traditional Didactic Lectures (DL); and radiographic interpretation skills of undergraduate dental students are suboptimal. Therefore, this study aims to assess whether utilizing BP to teach radiographic interpretation skills is an effective teaching methodology in Pakistan.

**Methods:**

This mixed-method study was conducted on final year dental students at Jinnah Medical and Dental College (JMDC). Two groups of students were utilized for this study, one taught by traditional DL and the other taught by BP for the same module. BP was conducted over six weeks. A post-module test was conducted in both groups. Additionally, the BP group completed a modified Community of Inquiry (CoI) survey tool and volunteered to discuss their experiences through a focused group discussion (FGD). Descriptive statistics were computed and independent sample t-test was used to analyse the difference between the scores of the two groups. Thematic analysis was performed for the qualitative data.

**Results:**

The mean post-test scores were found to be significantly higher in the BP group (61.0 ± 10.2) compared to the DL group (44.4 ± 12.3) (p = < 0.001, CI = 95%, Cronbach Alpha > 0.8). The mean scores for the modified CoI instrument were 4.0 ± 0.29 for the whole instrument; 4.25 ± 0.22 for Teaching Presence, 3.71 ± 0.23 for Social Presence and 3.97 ± 0.16 Cognitive Presence, with all three having a Cronbach’s alpha > 0.75. Thematic analysis revealed that BP students mutually agreed that BP method was beneficial with the appreciation of strong support from the facilitator. However, challenges like interrupted power supply and increased effort requirement from students were pointed out.

**Conclusion:**

Students taught radiographic interpretation skills with BP in comparison to DL had higher test scores and expressed a positive experience demonstrated via a modified CoI survey and FGD. Considering the encouraging results found, dental schools should incorporate BP in their teaching methodology and follow-up studies are needed to further support the use of BP as an effective teaching methodology in Dentistry.

**Supplementary Information:**

The online version contains supplementary material available at 10.1186/s12909-024-05062-5.

## Introduction

The didactic lecture (DL) is traditionally used to teach undergraduate dental students in Pakistan. However, due to its intrinsic weaknesses, DL has been unappealing to the digital learners. It is teacher-centered, often monotonous, with very limited avenues for the learners to engage with the content or with the peers [1]. On the contrary, the Blended Pedagogy (BP) is learner-centered, interactive and engaging. Learners interact in multiple dimensions, with, the teaching material; the peers (during the discussion session); and the facilitators, in a non-threatening, conducive environment.

BP is a widely adopted teaching strategy and is a synergistic blend of online and face-to-face learning, designed to benefit from strengths of both learning methods [2]. Literature supports the use of online discussion forums (ODF), which serves as a platform for communication in which students can participate in collaborative discussions with their peers and teachers [3, 4]. Not only does it promote deep learning, but it also aids teachers in identifying areas in which student lack proper understanding. Furthermore, utilizing ODF has also resulted in improved assessment scores, with students endorsing its use for positive learning outcomes [4, 5].

Interpretation of dental radiographs is a critical skill required in dental care providers, yet a recent study has found that dental students have suboptimal interpretation skills [6]. Although both dental undergraduate and post-graduate programs have experimented with BP in multiple dental disciplines, oral radiographic interpretation is predominantly taught via didactic lectures (DL) [7]. However, since interpretation of radiographs is visual in nature, BP can be utilized for learning the skill with a greater benefit to students critical thinking. In fact, in recent studies, using BP in undergraduate radiological education has also been reported to refine problem-solving capability and clinical knowledge, as well as improve learner satisfaction and confidence [8–10].

It is equally as important to also consider students’ perspective of utilizing BP as a learning tool [11]. While Focus group discussion (FGD) may be used to gain rich insight and a deeper understanding of the dynamics of student engagement during online discussion and face-to-face sessions, tools like Community of Inquiry (CoI) Survey instrument may provide objective and quantifiable data [12]. This tool is based on the CoI framework, built upon cognitive presence, teaching presence and social presence [13]. Its framework enables to create a deep and meaningful learning experience and the CoI survey instrument has been previously used to successfully measure learners’ engagement during large online courses [14].

When looking at dental education in the South Asian region such as Pakistan, most teaching in dentistry schools still relies on traditional methods. However, the recent emergence of digital education has led to some dental schools to rethink their teaching strategies [15]. Studies on the use of BP in dental education are also quite limited, with only a single study by Mirza et al. conducted in Karachi, Pakistan, reporting that dental students encountered difficulty in interpretation of radiographs during patient interactions [6].

### Rationale

Radiographic interpretation is an essential skill in undergraduate dental education, that contributes to the accuracy of diagnosis and therefore, treatment plan of the patient. However, it was observed repeatedly over the years, that the students were challenged with the interpretation of radiographs. Hence, this subject area was chosen for intervention and the current study.

BP is more than a single mode of instructional delivery and provides an individualized learning experience with convenience of time, pace and space to address the different preferences and needs of learners.

### Significance

Improved radiographic interpretation skill will consequently improve the students’ diagnostic accuracy, clinical practice and patient outcomes.

### Objectives

1. To compare if there is any difference in test scores of final year BDS students of Jinnah Medical and Dental College (JMDC), through DL and BP for radiographic interpretation.
2. To determine the effectiveness of BP approach administering the modified CoI survey tool in final year BDS students of JMDC.
3. To explore the perceptions of final year BDS students of JMDC regarding BP for radiographic interpretation.

## Methodology

### Setting

This study was conducted at JMDC, which is a private teaching institution, affiliated with Jinnah Sindh Medical University. JMDC currently follows a traditional teaching curriculum, for a four-year undergraduate dental program. Operative dentistry is one of the four subjects taught to final year dental students, which is usually a batch of 40–50 students. Within operative dentistry, radiographic interpretation makes up a relatively small, but important component. Previously, the radiographic interpretation module was conducted via DL, however for the purpose of our study, the use of BP for the same module was introduced. Hence the dental students taught using didactic lectures will be referred to as the didactic lecture group (DL group), and the dental students taught using blended pedagogy will be referred to as the BP group.

The planning and preparation for the study started in 2017, while the data was collected and analysed over five months in 2018.

### Study design

The current study has a mixed-method study design. The first part of the study was Quasi-experimental which included the BP and a post-test, which was followed by the second part of the study consisting of Focus Group Discussions (FGD) and a Community of Inquiry (CoI) survey, administered post-module.

### Study participants

#### Sampling technique and size

Universal sampling was employed for quantitative component of the study, i.e., the whole class of final year dental students was included in the study for both the DL (batch of 2017) and BP group (batch of 2018). For the qualitative component purposive sampling was done.

A total of 39 students agreed to participate from the DL group, whereas 43 students from the BP group agreed to participate in the study.

#### Inclusion criteria

All students of final year BDS, JMDC.

#### Exclusion criteria

Students who did not consent to participate in the study; were irregular in ODF; or missed the post-test; were excluded from the study.

Students unwilling to participate in the study were taught via the method planned for that year but were excluded from the study. The purpose and the methodology of the study was explained to the study participants and their written informed consent was taken.

### Blended pedagogy

Blended learning was conducted over six weeks of the module. After an orientation session for students, power point presentations, focusing oral radiographic interpretation, were uploaded on Google Drive followed by 2 relevant radiographs for online discussion using Google Docs. This was done in weeks 1, 2, 4 and 5. Face-to-face sessions were conducted in weeks 3 and 6 for clarification of students’ concepts. The uploaded power point presentations were the same ones as used for teaching the DL group.

### Online discussion forum

The Online discussion was conducted on Google Docs, in which the students were divided into four groups to maximize discussion contributions. Ground rules and clear instructions were provided to the students. The ODF was facilitated by the Principal Investigator. The facilitator was responsible to keep the students on track and clarify any misconceptions. Freedom of expression was ensured.

### Data collection tools and methods

Data were collected through post-test results, modified CoI survey instrument and an FGD. The findings from these three methods of data collection ensured methodological triangulation that added to the validity of our study.

#### Post-test

The post-test was developed by faculty of Operative Dentistry, at JMDC. Three content experts were invited to comment on the post-test and its keys, for validation and alignment with the module objectives and blue print. They responded on a proforma for appropriateness and relevance of question items. All the content experts were external.

The experts did not suggest any revision and none of the items was scored below 3 on a scale of 1 to 4 (least appropriate to most appropriate). The calculation of Content Validity Index (CVI) was not deemed necessary, because the content experts considered each item to be appropriate and relevant.

The post-test administered was the same for both the DL group and the BP group. It comprised of 15 radiographic images for interpretation. When conducted, the post test was run through display of images on-screen, with no physical copies handed over to the students. The answer sheets were collected back and the students were not allowed to use electronic devices during the post-test. So there was virtually no likelihood of contamination. The answers were checked using a scoring key, by a single assessor, who was not a part of study.

Post-test results were formative and remedial classes were arranged for students with low scores on post-test results for BP group. The results of DL group were used to compare with that of the BP group.

#### Modified community of inquiry survey

After the post-test the BP group were requested to complete the CoI survey. Permission to use the CoI framework and modification of the CoI survey tool was obtained through e-mail. The CoI was originally a 34-item survey instrument, possessing three categories of items, i.e. Teaching Presence, Social Presence and Cognitive Presence, with 13, 9 and 12 items, respectively (Additional File 1.). It had a 5-point scale (5-strongly agree through to 1-strongly disagree). After discussion within the research committee, we choose to remove two items as part of our instrument adaptation and the items in social presence reduced from nine to seven. While the CoI is a well validated instrument, the researchers decided to conduct a pilot for its final testing, after modification. The scoring, as is also shown in Table 2, was spread over three tiers: individual items, individual subscale and the whole instrument, using mean scores for each.

#### Focus group discussion (FGD)

After the post-test results were shared, students of the BP group were invited to participate in a focus group discussion (FGD) on a voluntary basis. The session was conducted at JMDC and was audio-recorded. Willingness to participate was considered as verbal consent. Written informed consent was taken prior to the FGD. Perceptions and experiences of students during the teaching of radiographic interpretation, using BP, were the focus of the FGD. Ground rules for participation in FGD were elaborated on, to ensure comfort and freedom of expression of the participants (Additional File 2). In total 11 participants were part of the FGD. Students were assigned codes to ensure anonymity.

The FGD was moderated by a medical educationist (other than the researcher) and the FGD guide was made available for him (Additional File 3). Trustworthiness of qualitative data was ensured by using pre-defined questions and respondent validation.

## Data analysis

All the data sets were kept anonymous and confidentiality was strictly ensured. Separate analysis was done to draw inferences from quantitative and qualitative data. Reporting of results was also done separately. This was later compared and synthesized.

For quantitative data analysis the post-test scores of both groups were entered in SPSS ver. 20. Descriptive statistics was generated including mean and standard deviation. Reliability coefficient –Cronbach’s Alpha, followed by item-total statistics were calculated for post- test scores of both the DL group and the BP group. Normal distribution of all data sets was confirmed through Shapiro-Wilk test. Therefore, the post-test scores of both groups were compared by independent sample t-test [16]. The confidence interval was set at 95% and the level of significance for the two tailed test was therefore alpha = 0.05.

Scores of modified CoI survey were also entered in SPSS. Reliability coefficient for CoI survey tool as a whole, and for each subscale (element) of CoI survey tool was calculated. Computation of item-total statistics was followed by descriptive statistics including mean and standard deviation for each subscale, as well as each item.

Qualitative data analysis began manually after the verbatim transcription of FGD. Respondent validation was done for verification of responses in the transcription; to minimize bias and to improve the credibility of results [17]. A priori codes were identified from literature; and from the data in relevance to the research question [18]. Coding was done independently by two researchers. An iterative approach was used. Identified themes were reviewed in conformation with the research question and theoretical framework. Subsequent to data saturation, inferences were drawn and consensus was reached [19].

## Ethical considerations

The study was approved by Ethical Review Committee of Jinnah Medical and Dental College (Ref. No. D-0102). The purpose of research was explained to the students and their written informed consent was taken. Participants had the right to refuse to participate in the study or withdraw from it at any time. Refusal or withdrawal did not affect the students in anyway. Data was accessible to researchers only, who ensured anonymity and confidentiality at all times. Study participants were assigned codes to mask their identity.

## Results

### Quantitative analysis

The DL group consisted of 39 students. While 43 students agreed to participate in the BP group, six had to be removed. These were the students who were either irregular in ODF participation or did not appear in the post-test. This left the BP group with 37 participants in all.

Characteristics and Test Scores analysis is shown in Table 1. In both groups more than 80% were females which is close to the representation of the gender distribution in the batches, however no statistically significant difference was found in the gender distribution of the two groups (*p* = 0.596).

**Table 1:** Comparison between didactic lecture group and blended pedagogy group

	Didactic Lecture Group (*n* = 39)	Blended Pedagogy Group (*n* = 37)	P-value	Effect size *d*_*Cohen*_
Male	7 (17.9%)	5 (13.5%)	0.596	> 1.3
Female	32 (82.1%)	32 (86.5%)		
Reliability of post test scores				
Number of test items = 15 (Cronbach alpha)	0.886	0.885	-	
Mean post test scores (mean ± SD)	44.4 ± 12.3	61.0 ± 10.2	< 0.001	

Reliability analysis for post-test revealed that Cronbach’s alpha was 0.88 for scores of both groups, implying that the set of questions had good internal consistency. Item-total statistics was calculated for each item on the post-test in both groups. Since the values of Cronbach’s alpha was above 0.8 for each item if deleted, in both groups, all items were retained (Additional Files 4 and 5).

The post-test scores of DL group and BP group were confirmed to have normality of data distribution in both data sets using the Shapiro-Wilk test. The maximum possible score for the post-test was 90. The mean post-test scores were found to be significantly higher in the BP group with a mean of 61.0 ± 10.2 compared to the DL group which had a mean of 44.4 ± 12.3 (p = < 0.001, CI = 95%). The effect size, *d*_*Cohen*_ was > 1.3.

Modified CoI survey tool was administered to 37 students of BP group. Reliability analysis and mean scores of the CoI survey tool is shown in Table 2. Overall sound internal consistency was demonstrated using Cronbach’s alpha for the whole instrument (0.88) along with each of its subscales especially showing high reliability for cognitive presence (0.873). All individual items for each subscale had a value of greater than 0.690 (see Additional file 6).

**Table 2:** Reliability analysis and mean scores of the modified community of inquiry (COI) survey

COI Survey Metrics	Mean ± SD	Cronbach’s Alpha if item deleted
Overall Survey items (*n* = 32) Cronbach’s Alpha: 0.88	4.03 ± 0.29	
**Individual Sub-scales**		
Total TP items (*n* = 13) Cronbach’s Alpha: 0.78	4.25 ± 0.22	
TP1	4.43 ± 0.50	0.765
TP2	4.46 ± 0.51	0.774
TP3	4.49 ± 0.65	0.754
TP4	4.62 ± 0.54	0.776
TP5	4.11 ± 0.61	0.749
TP6	4.24 ± 0.55	0.766
TP7	4.08 ± 0.60	0.787
TP8	4.24 ± 0.55	0.785
TP9	4.05 ± 0.88	0.750
TP10	3.92 ± 0.80	0.784
TP11	4.16 ± 0.65	0.786
TP12	4.03 ± 0.90	0.751
TP13	4.41 ± 0.55	0.791
Total SP items (*n* = 7) Cronbach’s Alpha: 0.75	3.71 ± 0.23	
SP1	3.62 ± 0.83	0.716
SP2	3.43 ± 1.01	0.699
SP3	3.92 ± 0.76	0.711
SP4	3.81 ± 0.88	0.691
SP5	3.57 ± 0.96	0.724
SP6	3.65 ± 0.82	0.751
SP7	4.14 ± 0.75	0.738
Total CP items (*n* = 12) Cronbach’s Alpha: 0.87	3.97 ± 0.16	
CP1	3.92 ± 0.64	0.871
CP2	3.76 ± 0.68	0.862
CP3	3.95 ± 0.88	0.854
CP4	4.00 ± 0.88	0.861
CP5	4.32 ± 0.63	0.858
CP6	3.92 ± 0.72	0.871
CP7	4.05 ± 0.57	0.862
CP8	3.95 ± 0.52	0.870
CP9	4.08 ± 0.49	0.865
CP10	3.73 ± 0.87	0.865
CP11	3.86 ± 0.79	0.852
CP12	4.14 ± 0.82	0.858

TP = Teaching Presence, SP = Social Presence, CP = Cognitive Presence

The overall mean score of CoI survey was calculated to be 4.03 ± 0.29. The mean scores for all the survey items ranged from a minimum of 3.43 to a maximum of 4.62 on the 5-point Likert agreement scale. The mean score of overall subscales was highest for teaching presence (4.25 ± 0.22) and lowest for social presence (3.71 ± 0.23).

### Qualitative analysis

The participants were generally vocal and expressive in the FGD, and shared their perceptions without hesitation. The responses towards BP were mostly positive. The synthesis of qualitative findings identified three themes (Table 3).

**Table 3:** Qualitative themes identified from focus group discussion

	Specific Theme
Theme 1	Experience with Blended pedagogy
Theme 2	Facilitator’s support
Theme 3	Challenges

#### Theme 1: Experience with blended pedagogy

BP was well-accepted by most students. For most of the students it was a new, interesting and enriching experience which they cherished *“Looking forward to blended pedagogy!”* and *“It was a new thing for me to learn.”*

One of the participants enjoyed the experience so much that she suggested *“…These activities should be a part of other modules.”*

Overall, the students were satisfied with the design of the BP, systematic organization of the content and the resources shared with them. One of the students commented:

It was well organized and instructions were clear.

Relevance of the content was appreciated by most of the students. For example, a participant stated:

The design and the content of the module were very relevant to our syllabus, and it helped us….

Another student expressed:

It was interesting in a way that whatever we read or searched, we were using it in our clinical rotations.

All the participants agreed that the content delivered to them provided the required knowledge base. Students also discussed that the content provided, gradually enabled them to acquire radiographic interpretation skills of cases with higher level of complexity. One student verbalized *“…it was initially basic and then with the time it became complicated (advance).”*

All the students believed there was sufficient time for them to read, search and discuss the answers. For instance, a participant articulated that *“I think the flow was okay and the pace matched ours.”*

All students expressed that they improved their radiographic interpretation skills and hence were able to reach a diagnosis with justification. They shared that they had to search a lot to reach to the correct answer and in the process, they gained a lot of knowledge leading to improved problem-solving abilities. One student mentioned that *“If we want to excel in our field, we should not rely on our bookish knowledge only. It (BP approach) also developed a habit of searching into multiple sources for the single topic which helped in opening our minds and it also helped in making our concepts much clearer.”* Another student added that *“In the whole process I did a lot of critical thinking.”*

One of the students remarked that the ODF was designed in such a way that they had to use their capabilities to combine knowledge from different sources to unravel the problems presented to them, with reasoning, and also said *“…It allowed me to come up with solutions for difficult scenarios and it also taught me self-directed learning.”*

One student quoted *“I am able to solve problems on my own. I am less dependent on others.”*

All FGD participants agreed that their scores on the test of radiographic interpretation had improved with the use of BP, compared to their scores on other modules using DL. The students also mentioned that they were not stressed when they took the test because they had prepared well during online discussions. *“I would not have managed to pass the test if I wouldn’t have attended the online discussion,”* a student stated. Another student endorsed the same idea by saying *“I am an average student, but my scores improved. I was among the top 5 students in this test.”*

Students also enjoyed the flexibility of time and place that BP provides for individualized learning experiences. One student shared *“I was out of the country for five days and I could access the online discussion from over there and I completed my task in due time.”* A student reinforced the point of view *“… I could work in night-time and that helped me.”*

They also affirmed that the ODFs were engaging and described the experience by remarking *“… it kept me interested in it.”.* Opportunity of reflection, record of the discussion thread, and ability to review it later, were also valued by all students, with a student commenting that *“…We can review it any time*”. Gaining insight about other students’ perspectives during the ODF was also appreciated by most of the participants, since it was not possible during DL.

#### Theme 2: Facilitator’s support

Students expressed a positive attitude towards facilitator’s support and valued the availability and timely response of the facilitator. One of the students mentioned that *“The facilitator was always there.”* Another student substantiated by commenting:

…It was easy for me to approach her and ask about my queries.

The guidance and focus provided by the facilitator was well-acknowledged by all the students. One student shared *“…The facilitator helped us get back on track and helped me to look at the problem with another angle…”.* A student supported the comment by adding *“…She guided us towards our destination, but we reached our destination ourselves.”*

One participant expressed “*The facilitator’s role was quite motivating and her attitude encouraged me to show more interest.”* All the FGD participants agreed that their misconceptions were clarified during the ODF and face-to-face sessions.

All the students expressed their satisfaction with the learning environment. They shared that they were free to express their views without the fear of being criticized or degraded by anyone. They also mentioned that they could easily communicate their difference of opinion as one participant said, *“I really enjoyed disagreeing with my classmates and group members.”*This was echoed in a statement from another participant *“… Yes, it was a very friendly environment. We were very comfortable.”* Another participant endorsed it, *“Initially, I was very hesitant before replying or commenting on someone’s post. The facilitator created a very good environment that made us come to the forefront to answer.”* Overall, students believed that BP was beneficial for students who were shy and less vocal during face-to-face sessions.

#### Theme 3: Challenges

Students were not used to the idea of asynchronous discussions or searching for answers themselves, since this was their first exposure to BP with one student expressing that *“…if someone teaches me my learning becomes better.”* They voiced that BP was more demanding on time and effort. For example, a student said *“…We had to search ourselves, we had to put in time and then… the typing…”.* With reference to technical difficulties, only one student had a problem with logging in as he commented, *“I faced certain difficulties in the initial stages. I had an issue with the access and because it was new, I could not understand where to type and how to type.”* One student also complained of power failures for which she had to manage her time accordingly *“…The issue that I faced most was, the area where I live in, load shedding (power shortage) was a big problem.”*

## Discussion

### Effect of blended pedagogy on assessment scores

Based on our literature search, our study is the first of its kind to assess the effectiveness of BP in radiographic interpretation skills of dental students in Pakistan. Our study found that using BP led to significantly higher post-test scores than students who were taught the same content using DL (*p* < 0.05). Limited studies are available in the literature which have particularly assessed student performance scores after implementing BP for dental radiographic interpretation skills. One study conducted at an undergraduate medical school in Australia, found results similar to ours, since they concluded that students receiving a combination of traditional learning and e-learning, had improved knowledge and skills in X-ray interpretation and significantly higher assessment scores, than students who did not receive e-learning [9]. Other studies found similar results by demonstrating higher test scores in radiographic interpretation via BP for learning compared to traditional lectures [20–22]. Interestingly, a study done by Tan et al. found that although student grades were higher in the group utilizing BP compared to the DL alone group, no statistically significant difference was observed between exam grades of students who opted for e-learning alone versus BP [23]. In contrast to our study findings, Nkenke et al. reported their randomized controlled trial in which students who participated in technology-enhanced learning for a theoretical radiological science course, had a performance score similar to students who had attended traditional lectures, and that the students in their study preferred conventional lectures as the basis for university education [24]. Similarly, Ketelsen et al. also found no significant difference between test results in groups utilizing different modes of content delivery such as lectures, printed text or just digital, in radiological anatomy lessons [25].

The significantly higher test scores achieved by BP group, could be due to the fact that the students felt empowered using the power point presentations with radiographic images at the time and pace that was workable for them [26]. Furthermore, active engagement with the peers and radiographic images through ODF, strengthened their concepts and learning [27]. The students’ voices during the FGD, corroborated these explanations.

### Modified community of inquiry, CoI survey

The Modified CoI has been successfully used for assessing perceptions with BP [28–30]. However, we believe our study is the first of its kind which has used the Modified CoI tool to assess dental student perceptions regarding BP. A recent study conducted in nursing students by Siah et al., in which they used a 5-point scale in their CoI survey to assess effectiveness of BP in clinical nursing skills, demonstrated findings which reflected ours, since they found the highest mean score of 4.11 for teaching presence and lowest mean score of 3.76 for social presence [29].

The mean scores of subscales and whole CoI instrument verified the theoretical underpinnings of CoI framework. The highest mean score for teaching presence could be elucidated considering the fact that ground rules, expectations and guidelines were provided to the students before the beginning of the module. Facilitator’s availability and prompt response was highly appreciated by the students, as highlighted in FGD as well. High scores on social presences explain that the students acknowledged being a part of online community, especially when contributing in the ODF, without feeling threatened. Improved critical thinking and problem solving skills experienced by students as a result of blended pedagogy, explain the mean scores of cognitive presence.

The CoI reliability analysis in our study, for the overall CoI instrument and each of its subscales, revealed values of Cronbach’s alpha to be in the range of 0.75–0.88 (moderately reliable to highly reliable), which is similar to a prior study by Mills et al. (0.70); although lower than the study findings by Siah et al. (0.90–0.96) [28, 29]. Hence, it can be inferred that the CoI scale and its subscales have high inter-correlations leading to sound internal consistency [31]. It can thus be stated that the CoI survey tool, that was used in our study, is a reliable method to assess the existence of CoI and its elements in BP modules.

### Student experiences with blended pedagogy via online discussion forums

For the ODF, the findings from our study showed that overall, the students appreciated the BP module due to its systematic approach along with clear instructions, supportive and conducive learning environment and improvement in radiological interpretation and critical thinking skills. The students also mentioned a unique benefit of the ODF was the ability to also learn from reading other students’ comments, hence adding to the teaching aspect of the CoI. They mentioned that they saw similar cases in their clinical rotation, and that the radiographs discussed were presented in a logical sequence, with an increasing level of complexity helped foster critical thinking. The BP study in radiological interpretation skills by Salajegah et al. also identified that students appreciated the online resource and found it to be clinically relevant [9]. In our study, most of the students were satisfied with the pace and flow of online content delivery. This was probably since the power-point presentations were easy to understand, with no barriers in time and space to utilize them, hence providing students flexibility in their learning. Moreover, students appreciated that they had enough time to read and think before they constructed their comments during the ODF, which further adds to the cognitive presence of the CoI framework.

Several other studies utilizing BP, reported similar responses of students, who enjoyed the learning content and organization of such pedagogy [21, 32]. In fact, Morton et al. stated that the logical structure of content that builds from simple to complex ideas improves students understanding of the topic [33]. Furthermore, Tan et al. found a significant correlation between exam grades and students’ perception of the quality of e-content presented in dental radiology [23]. Regarding the use of BP in radiographic interpretation skills in particular, a recent study by Pereira et al. also found that the use of BP led to a predominately positive impact on students’ confidence in the interpretation of radiographic findings [34].

Student contentment with the learning environment and the facilitator’s support in our study, could be attributed to emphasis on ground rules and expectations for the ODF, done by the facilitator before beginning it, leading to a respectful and non-judgmental environment. This was supported in another study which found that students’ commitment and engagement in ODFs can be fostered with faculty support, teaching exciting topics, and providing sufficient time to understand new learning terrain [35]. Students were comfortable communicating differing perspectives, which also aligns with the social presence aspect of the CoI. Cassum et al. linked student’s motivation to perform better with teacher’s involvement [32]. In fact, previous studies have also stated that the facilitator is responsible to encourage students, to think critically, by using open-ended questions to probe them; and by providing timely and constructive feedback during ODFs, hence, confirming teaching presence in their study [36, 37].

Students in our study believed that their radiographic interpretation skills, critical thinking and problem-solving abilities developed during the BP module, leading to reduced dependence on others and self-directed learning. This verifies the cognitive presence during the module, in alignment with the CoI framework. The underlying reason could be that instead of spoon feeding, students received encouragement and impetus to solve problem by themselves and take the path of self-study.

### Challenges with blended pedagogy

The challenges we identified during the BP module included disruption of electric supply, and one student temporarily facing technical issue with logging in. In a low-middle income country such as Pakistan, power outages are common and may cause difficulty to students and teachers alike in utilizing online educational platforms. Such difficulty has also been reported in previous studies using BP in Pakistan [32, 38]. Hence, operational challenges associated with the use of the e-components of BP, may limit the program’s ability to deliver optimally. Furthermore, a complaint of increased time and effort was also reported by students. However, adjustment to BP and onus of responsibility of self-study, that comes alongside the flexibility of time and pace, was challenging for some students.

Moreover, for a BP program to be successful, it is imperative that the learners are self-motivated, can manage time effectively and can take the responsibility of their own learning [39]. Faculty members also agree that utilizing BP increases their workload and changes the classroom dynamics [40]. In addition, slow typing speed, which is a challenge itself, was reported by some students as a barrier resulting in more time spent answering a question compared to face-to-face lectures. A qualitative study by Cassum et al. which reported student experiences and perceptions regarding BP in Pakistan, also found that students in their study remarked that a change in the learning methods is difficult to accept, although they were convinced that they will adjust to the change in time [32]. And most importantly, using BP reduces social interaction, which was also reflected in our study by relatively low social presence scores [40].

### Strengths and limitations

To the best of our knowledge, this is the first study of its kind in the South Asian region, which is assessing the effectiveness of BP in radiographic interpretation skills among dental students. It is also the first study of its kind which has utilized Google Docs for ODF and the CoI survey tool in order to assess dental students’ perceptions. Furthermore, this study was a mixed method study which gave us an opportunity to support our findings in more than one way (via triangulation), hence providing extensive insight which compensates for deficiencies in any one method of assessment.

The biggest limitation of this study is that it is a single-centre study with a relatively small sample size, hence results may not be very generalizable. Secondly, the two cohorts of the BP group and DL group may be different in that the BP group students may have been more motivated to perform better than the DL group. In the absence of baseline variables to compare between the two groups, this could not be ascertained. Additionally, the facilitator might have introduced bias by teaching more enthusiastically along with possibly causing a Hawthorne effect based on expecting improved performance and positive student feedback. Lastly, possible contamination of questions being leaked exists because the same post-test was used for DL group as well as BP group.

### Directions for future research

The favourable results of this study encourage more studies to be conducted in multiple centres, with larger sample sizes and broader coverage of dental curriculum.

## Conclusion

Our study showed that students taught radiographic interpretation skills with BP in comparison to DL had significantly higher test scores (effect size, *d*_*Cohen*_ > 1.3). Moreover, the students found it more effective (in terms of engagement and satisfaction with BP), demonstrated via a modified CoI survey. Positive perceptions and experiences were shared through FGD. Considering the encouraging results found, dental schools have evidence to incorporate BP in their repertoire of teaching methodologies. However, follow-up studies are needed to further support the use of BP as an effective teaching methodology across other content areas of Dentistry.

### Electronic supplementary material

Below is the link to the electronic supplementary material.

**Supplementary Material 1:** Modified CoI survey Instrument (post module evaluation)

**Supplementary Material 2:** Ground rules for Focus Group Discussion

**Supplementary Material 3:** Focus Group Discussion Guide

**Supplementary Material 4:** Item total statistics of post-test scores of Didactic Lecture Group

**Supplementary Material 5:** Item total statistics of post-test scores of Blended Pedagogy Group

**Supplementary Material 6:** Item total statistics of modified CoI survey and its sub-scales (Blended Pedagogy Group)

## Data Availability

The datasets used and/or analysed during the current study are available from the corresponding author on reasonable request.

## Abbreviations

BDS: Bachelor of Dental Surgery
BP: Blended Pedagogy
CoI: Community of Inquiry
DL: Didactic Lectures
FGD: Didactic Lectures
JMDC: Jinnah Medical and Dental College
ODF: Online Discussion Forum
