# Supplementary material for: Effectiveness of blended pedagogy for radiographic interpretation skills in operative dentistry - a comparison of test scores and student experiences at an undergraduate dental school in Pakistan
Source: BMC Med Educ. 2024 Jan 22;24:80. doi: 10.1186/s12909-024-05062-5 (PMC10804605; doi:10.1186/s12909-024-05062-5)
Supplement: Supplementary file 1 — Supplementary Material 1: Modified CoI survey Instrument (post module evaluation) [file 12909_2024_5062_MOESM1_ESM.docx]

## Additional File 1- Modified CoI survey Instrument (post module evaluation)

| **Name :** | **Roll. No. :** |
| --- | --- |
| **Contact number:** | **Module: Radiological Interpretation Skills** |
| **Email:** | |

| You are requested to please fill out the form honestly. | Please circle the most appropriate response  1 = strongly disagree, 2 = disagree, 3 = neutral, 4 = agree, 5 = strongly agree | | | | |
| --- | --- | --- | --- | --- | --- |
| **Teaching Presence** |  |  |  |  |  |
| Design and organization |  |  |  |  |  |
| 1. The instructor clearly communicated important module topics. | 1 | 2 | 3 | 4 | 5 |
| 2. The instructor clearly communicated important module goals. | 1 | 2 | 3 | 4 | 5 |
| 3. The instructor provided clear instructions on how to participate in learning activities of this module. | 1 | 2 | 3 | 4 | 5 |
| 4. The instructor clearly communicated important due dates/time frames for learning activities in this module. | 1 | 2 | 3 | 4 | 5 |
| Facilitation |  |  |  |  |  |
| 5. The instructor was helpful in identifying areas of agreement and disagreement on topics that helped me to learn. | 1 | 2 | 3 | 4 | 5 |
| 6. The instructor was helpful in guiding the class towards  understanding topics in this module in a way that helped me clarify my thinking. | 1 | 2 | 3 | 4 | 5 |
| 7. The instructor helped to keep module participants engaged and participating in productive dialogue. | 1 | 2 | 3 | 4 | 5 |
| 8. The instructor helped keep the module participants on task in a way that helped me to learn. | 1 | 2 | 3 | 4 | 5 |
| 9. The instructor encouraged participants to explore new concepts in this module. | 1 | 2 | 3 | 4 | 5 |
| 10. Instructor actions reinforced the development of a sense of community among participants. | 1 | 2 | 3 | 4 | 5 |
| Direct Instruction |  |  |  |  |  |
| 11. The instructor helped to focus discussion on relevant issues in a way that helped me to learn. | 1 | 2 | 3 | 4 | 5 |
| 12. The instructor provided feedback that helped me understand my strengths and weaknesses relative to the module’s goals and objectives. | 1 | 2 | 3 | 4 | 5 |
| 13. The instructor provided feedback in a timely fashion. | 1 | 2 | 3 | 4 | 5 |
| **Social Presence** |  |  |  |  |  |
| Affective expression |  |  |  |  |  |
| 14. Online or web-based communication is an excellent medium for social interaction. | 1 | 2 | 3 | 4 | 5 |
| Open Communication |  |  |  |  |  |
| 15. I felt comfortable conversing through the online medium. | 1 | 2 | 3 | 4 | 5 |
| 16. I felt comfortable participating in the module discussions. | 1 | 2 | 3 | 4 | 5 |
| 17. I felt comfortable interacting with other module participants. | 1 | 2 | 3 | 4 | 5 |
| Group Cohesion |  |  |  |  |  |
| 18. I felt comfortable disagreeing with other module participants while still maintaining a sense of trust. | 1 | 2 | 3 | 4 | 5 |
| 19. I felt that my point of view was acknowledged by other module participants. | 1 | 2 | 3 | 4 | 5 |
| 20.Online discussions help me to develop a sense of collaboration. | 1 | 2 | 3 | 4 | 5 |
| **Cognitive Presence** |  |  |  |  |  |
| Triggering Event |  |  |  |  |  |
| 21. Problems posed increased my interest in module issues. | 1 | 2 | 3 | 4 | 5 |
| 22. Module activities piqued my curiosity. | 1 | 2 | 3 | 4 | 5 |
| 23.I felt motivated to explore content related questions. | 1 | 2 | 3 | 4 | 5 |
| Exploration |  |  |  |  |  |
| 24. I utilized a variety of information sources to explore problems posed in this module. | 1 | 2 | 3 | 4 | 5 |
| 25. Brainstorming and finding relevant information helped me resolve content related questions. | 1 | 2 | 3 | 4 | 5 |
| 26. Online discussions were valuable in helping me appreciate different perspectives. | 1 | 2 | 3 | 4 | 5 |
| Integration |  |  |  |  |  |
| 27. Combining new information helped me answer questions raised in module activities. | 1 | 2 | 3 | 4 | 5 |
| 28. Learning activities helped me construct explanations/solutions. | 1 | 2 | 3 | 4 | 5 |
| 29. Reflection on module content and discussions helped me understand fundamental concepts in this class. | 1 | 2 | 3 | 4 | 5 |
| Resolution |  |  |  |  |  |
| 30. I can describe ways to test and apply the knowledge created in this module. | 1 | 2 | 3 | 4 | 5 |
| 31. I have developed solutions to module problems that can be applied in practice. | 1 | 2 | 3 | 4 | 5 |
| 32. I can apply the knowledge created in this module to my work or other non-class related activities. | 1 | 2 | 3 | 4 | 5 |
