# Supplementary material for: Effectiveness of blended pedagogy for radiographic interpretation skills in operative dentistry - a comparison of test scores and student experiences at an undergraduate dental school in Pakistan
Source: BMC Med Educ. 2024 Jan 22;24:80. doi: 10.1186/s12909-024-05062-5 (PMC10804605; doi:10.1186/s12909-024-05062-5)
Supplement: Supplementary file 2 — Supplementary Material 2: Ground rules for Focus Group Discussion [file 12909_2024_5062_MOESM2_ESM.docx]

## Additional File 2- Ground rules for Focus Group Discussion

| **Ground Rules to be followed during Focus Group Discussion** |
| --- |
| 1. Please make sure not to discuss the details of Focus Group Discussion with anyone, or disclose any participant’s identity, after leaving the discussion site. |
| 1. Kindly sign the informed consent and ensure confidentiality. |
| 1. Please respect others and take turns in talking. |
| 1. Interruptions will not be allowed/ acceptable. |
| 1. Please make sure not to humiliate, single out, point out, disapprove, criticize, name-call or make any participant uncomfortable. Allow other participants to express their opinion without the fear of being disregarded, derogative or belittled. |
| 1. Please cooperate with the moderator and follow the instructions/ guidelines being provided by the moderator from time to time. |
