# Supplementary material for: Effectiveness of blended pedagogy for radiographic interpretation skills in operative dentistry - a comparison of test scores and student experiences at an undergraduate dental school in Pakistan
Source: BMC Med Educ. 2024 Jan 22;24:80. doi: 10.1186/s12909-024-05062-5 (PMC10804605; doi:10.1186/s12909-024-05062-5)
Supplement: Supplementary file 3 — Supplementary Material 3: Focus Group Discussion Guide [file 12909_2024_5062_MOESM3_ESM.docx]

## Additional File 3- Focus Group Discussion Guide

| **Focus Group Discussion guide for the Moderator** |
| --- |
| Moderator: Welcome to the Focus Group Discussion. This session will last about 60-90 minutes and will be audio-recorded. I hope that the purpose of the research is clear to you. I also want to take this opportunity to reassure you of the anonymity and confidentiality of data. Researchers, however, will have access to the data. Please read and sign the informed consent if you agree to proceed with the FGD. Your participation in this FGD is voluntary and you may choose not to answer any question that you are not comfortable with. You can withdraw from the FGD or study at any time. Attached to the informed consent form is a list of GROUND RULES you are requested to abide by during the session. You can withdraw from the FGD or research at any time without any untoward consequences to you. As you have been told that the confidentiality needs to be maintained during the study, please be sure not to discuss the content of the FGD with anyone or disclose any participant’s identity, once the session is over. |
| Following PROBES/ TRIGGERS may be needed:   1. Neutral Open-ended questions 2. Elaborative and clarifying questions including re-stating of the information provided. 3. Verbal fillers e.g. I see…, hummm….. 4. Non-verbal communication (Nodding) etc. |
| I am Dr. ----------, and I will be moderating this session.  I now request you to please introduce yourself one by one. We will begin from my right-hand side. |
| ICE- BREAKING QUESTION |
| 1. Please share your experiences regarding Blended Pedagogy (BP).   Probes: Could you please relate to a situation where you faced difficulty? Was there any situation where you felt BP was advantageous/ beneficial? |
| 1. How comfortable were you with the online discussions/ face-to face sessions?   Probe: environment, freedom of expression |
| 1. What was the facilitator’s/ teacher’s role during discussions?   Probe: Guided, brought us on track, clarified misconceptions, timely response, encouraging, engaging etc.  I see….. Hmmm…. |
| 1. How was the design and organization of the content?   Probe: Flow, pace, clear instructions etc. |
| 1. What do you think of activities during the module?   Probe: How helpful were the activities in arousing interest, motivation to learn more?  How far did the activities help you resolve issues with interpretation of radiographs? |
| 1. In your opinion, what was the effect of Blended Pedagogy (BP) on your scores? Did the scores improve? |
| Moderator: The session has now come to an end. We are very thankful for your cooperation. You are welcome to add or ask anything pertinent to this FGD. We will contact you for another discussion session if any further queries arise. The research will be shared with you when it is published. Thank you again for taking time out from your busy schedule. |
