# Supplementary material for: Effectiveness of blended pedagogy for radiographic interpretation skills in operative dentistry - a comparison of test scores and student experiences at an undergraduate dental school in Pakistan
Source: BMC Med Educ. 2024 Jan 22;24:80. doi: 10.1186/s12909-024-05062-5 (PMC10804605; doi:10.1186/s12909-024-05062-5)
Supplement: Supplementary file 4 — Supplementary Material 4: Item total statistics of post-test scores of Didactic Lecture Group [file 12909_2024_5062_MOESM4_ESM.docx]

## Additional File 4- Item total statistics of post-test scores of Didactic Lecture Group

| **Item-Total Statistics post-test scores of DL group** | | | | | |
| --- | --- | --- | --- | --- | --- |
| **Items** | **Scale Mean if Item Deleted** | **Scale Variance if Item Deleted** | **Corrected Item-Total Correlation** | **Squared Multiple Correlation** | **Cronbach's Alpha if Item Deleted** |
| 1 | 41.5449 | 138.947 | .356 | .534 | .886 |
| 2 | 40.8974 | 126.160 | .701 | .595 | .872 |
| 3 | 42.3782 | 135.059 | .476 | .583 | .882 |
| 4 | 42.0705 | 136.358 | .573 | .488 | .879 |
| 5 | 40.7115 | 135.147 | .496 | .545 | .881 |
| 6 | 41.2628 | 131.220 | .618 | .560 | .876 |
| 7 | 41.4423 | 132.669 | .616 | .578 | .876 |
| 8 | 40.5064 | 131.258 | .674 | .633 | .874 |
| 9 | 41.4936 | 134.620 | .478 | .399 | .882 |
| 10 | 41.4936 | 129.646 | .589 | .507 | .877 |
| 11 | 41.4167 | 129.810 | .509 | .601 | .881 |
| 12 | 41.6346 | 125.082 | .585 | .664 | .878 |
| 13 | 41.5705 | 139.707 | .330 | .593 | .887 |
| 14 | 41.8782 | 128.750 | .762 | .742 | .870 |
| 15 | 41.8910 | 133.463 | .553 | .584 | .879 |
