# Supplementary material for: Effectiveness of blended pedagogy for radiographic interpretation skills in operative dentistry - a comparison of test scores and student experiences at an undergraduate dental school in Pakistan
Source: BMC Med Educ. 2024 Jan 22;24:80. doi: 10.1186/s12909-024-05062-5 (PMC10804605; doi:10.1186/s12909-024-05062-5)
Supplement: Supplementary file 5 — Supplementary Material 5: Item total statistics of post-test scores of Blended Pedagogy Group [file 12909_2024_5062_MOESM5_ESM.docx]

## Additional File 5- Item total statistics of post-test scores of Blended Pedagogy Group

| **Item-Total Statistics of post-test scores of BP group** | | | | | |
| --- | --- | --- | --- | --- | --- |
| **Items** | **Scale Mean if Item Deleted** | **Scale Variance if Item Deleted** | **Corrected Item-Total Correlation** | **Squared Multiple Correlation** | **Cronbach's Alpha if Item Deleted** |
| 1 | 57.34 | 93.945 | .445 | .624 | .882 |
| 2 | 56.72 | 95.508 | .554 | .715 | .879 |
| 3 | 57.64 | 88.926 | .628 | .626 | .875 |
| 4 | 57.62 | 91.103 | .519 | .537 | .880 |
| 5 | 56.89 | 94.863 | .444 | .520 | .882 |
| 6 | 56.49 | 92.354 | .537 | .555 | .879 |
| 7 | 57.19 | 96.533 | .388 | .432 | .884 |
| 8 | 56.47 | 95.735 | .511 | .588 | .880 |
| 9 | 56.76 | 89.564 | .672 | .682 | .873 |
| 10 | 56.64 | 94.453 | .487 | .529 | .881 |
| 11 | 56.41 | 89.901 | .526 | .579 | .880 |
| 12 | 56.85 | 82.012 | .743 | .788 | .868 |
| 13 | 55.64 | 93.453 | .549 | .642 | .878 |
| 14 | 57.91 | 86.914 | .608 | .591 | .876 |
| 15 | 57.27 | 90.369 | .639 | .677 | .874 |
