# Supplementary material for: Effectiveness of blended pedagogy for radiographic interpretation skills in operative dentistry - a comparison of test scores and student experiences at an undergraduate dental school in Pakistan
Source: BMC Med Educ. 2024 Jan 22;24:80. doi: 10.1186/s12909-024-05062-5 (PMC10804605; doi:10.1186/s12909-024-05062-5)
Supplement: Supplementary file 6 — Supplementary Material 6: Item total statistics of modified CoI survey and its sub-scales (Blended Pedagogy Group) [file 12909_2024_5062_MOESM6_ESM.docx]

**Additional File 6****- Item total statistics of modified CoI survey and its sub-scales (Blended Pedagogy Group)**

| **Item total statistics (CoI)** | | | | |
| --- | --- | --- | --- | --- |
|  | Scale Mean if Item Deleted | Scale Variance if Item Deleted | Corrected Item-Total Correlation | Cronbach's Alpha if Item Deleted |
| TP01 | 124.6216 | 117.353 | .337 | .886 |
| TP02 | 124.5946 | 118.192 | .257 | .887 |
| TP03 | 124.5676 | 112.697 | .590 | .882 |
| TP04 | 124.4324 | 116.808 | .354 | .886 |
| TP05 | 124.9459 | 114.386 | .496 | .884 |
| TP06 | 124.8108 | 117.158 | .322 | .886 |
| TP07 | 124.9730 | 117.583 | .259 | .887 |
| TP08 | 124.8108 | 119.769 | .101 | .890 |
| TP09 | 125.0000 | 112.167 | .447 | .884 |
| TP10 | 125.1351 | 117.898 | .159 | .890 |
| TP11 | 124.8919 | 116.821 | .289 | .887 |
| TP12 | 125.0270 | 112.694 | .408 | .885 |
| TP13 | 124.6486 | 116.901 | .342 | .886 |
| SP01 | 125.4324 | 114.863 | .322 | .887 |
| SP02 | 125.6216 | 111.908 | .388 | .886 |
| SP03 | 125.1351 | 115.176 | .339 | .886 |
| SP04 | 125.2432 | 110.578 | .538 | .882 |
| SP05 | 125.4865 | 111.146 | .456 | .884 |
| SP06 | 125.4054 | 112.581 | .458 | .884 |
| SP07 | 124.9189 | 113.688 | .438 | .884 |
| CP01 | 125.1351 | 116.176 | .340 | .886 |
| CP02 | 125.2973 | 114.548 | .428 | .885 |
| CP03 | 125.1081 | 109.488 | .598 | .881 |
| CP04 | 125.0541 | 111.553 | .480 | .883 |
| CP05 | 124.7297 | 111.758 | .689 | .880 |
| CP06 | 125.1351 | 113.620 | .464 | .884 |
| CP07 | 125.0000 | 113.833 | .580 | .882 |
| CP08 | 125.1081 | 116.599 | .389 | .885 |
| CP09 | 124.9730 | 115.860 | .487 | .884 |
| CP10 | 125.3243 | 111.336 | .500 | .883 |
| CP11 | 125.1892 | 111.158 | .572 | .881 |
| CP12 | 124.9189 | 108.910 | .681 | .879 |

TP= Teaching Presence, SP= Social Presence, CP= Cognitive Presence
